# The impact of environmental parameters on microcystin production in dialysis bag experiments

**DOI:** 10.1038/srep38722

**Published:** 2016-12-09

**Authors:** Liqiang Xie, Richard R. Rediske, Nadia D. Gillett, James P. O’Keefe, Brian Scull, Qingju Xue

**Affiliations:** 1 State Key Laboratory of Lake Science and Environment, Nanjing Institute of Geography and Limnology, Chinese Academy of Sciences, 73 East Bejing Road, Nanjing 210008, China; 2 Annis Water Resources Institute, Grand Valley State University, 740 West Shoreline Drive, Muskegon, MI 49441, USA; 3 University of Chinese Academy of Sciences, Beijing 100049, China

## Abstract

It is important to understand what environmental parameters may regulate microcystin (MC) production and congener type. To determine if environmental conditions in two hydraulically connected lakes can influence MC production and congener ratios, we incubated dialysis bags containing phytoplankton from mesotrophic/eutrophic Muskegon Lake into hypereutrophic Bear Lake (Michigan, USA) and vice versa. Strong cyanobacteria growth was observed in all dialysis bags with Bear Lake phytoplankton in July and August. Phytoplankton communities were dominated by *Aphanizomenon aphanizomenoides*, *Microcystis wesenbergii*, *Limnothrix redekei*. MC concentrations were correlated with *M. wesenbergii* and *A. aphanizomenoides* biovolume. MC concentrations in bags incubated in the Muskegon Lake with Bear Lake water were significantly higher than the other bags. The higher light intensity and total nitrogen concentration may have caused the increase of MC production. The MC-LR/MC-RR ratios varied with sample origin but not with lake of incubation, indicating that physical environmental factors (water temperature and turbidity) were not the reasons for different toxin production ratios. Differences in total phosphorus concentrations might be one reason for the dissimilarity of the MC-LR/MC-RR ratio between the two lakes. The higher light intensity and NO_3_-N concentration in Muskegon Lake are two factors contributing to an increase of MC production.

Toxic cyanobacterial blooms occur frequently in eutrophic fresh waters worldwide^1^. Recent increases in cyanobacterial blooms are a cause for concern because they are known to produce a wide variety of toxins. Cyanotoxins can threaten the supply of drinking water and fisheries-related food supplies^2^,^3^. In addition, the toxins can accumulate in organisms and be transferred via aquatic food webs, presenting potential risks to human health^4^. The most widespread cyanotoxins in the environment is microcystin (MC), and more than 100 MC congeners have been identified from cyanobacterial blooms and cultures^5^. Congener type is a very important consideration in a bloom because the dominance of one congener over another will affect the toxicity^6^. Microcystin-LR (MC-LR) is the most common congener in freshwater^7^, also is one of the most hepatotoxic congeners^8^. Mouse assays indicated that the MC-LR and MC-LA variants were equally toxic, but were 12 times more toxic than another common congener MC-RR. Water bodies with regular dominance of specific taxa are likely to exhibit characteristic patterns of microcystin variants^9^. Many studies have focused on environmental parameters, such as water temperature^10^,^11^, phosphorus and nitrogen^12^, stoichiometric ratio of available nitrogen to phosphorus^13^, and pH^14^ on total microcystin production. However, only a few studies evaluated the effect of environmental parameters on the ratio of MC congeners and their relative abundances. In Anabaena 90, Rapala *et al*.^15^ suggested that the different MC variants were affected by temperature. In *Microcystis aeruginosa* HUB 5-2-4, Hesse and Kohl^16^ indicated that congener type was affected by light intensity and nutrient supply. In *Planktothrix agardhii*, the MC-LR and MC-RR ratio was affected by photon irradiance^17^ or amino acid availability (leucine and arginine)^18^. Monchamp *et al*.^13^ suggested that total nitrogen, water temperature, ammonium and dissolved organic nitrogen influenced the cyanobacterial community structure, which in turn resulted in differences in the dominant MC congener and the overall toxicity. Puddick *et al*.^7^ found the relative abundance of arginine-containing MC decreased as nitrate was depleted from the culture medium, indicating nitrogen played an important role in modulating the toxicity of *Microcystis*. Most of these studies examine the influence of environmental factors on the MC congeners in the lab and evidence for this influence in natural systems is limited. Knowledge on how environmental variables regulate MC congener abundance will assist in predicting the periods of greatest risk to human users exposed to these toxins^7^. It is important to understand what environmental parameters may regulate MC production but to determine what factors influence the variants of MC congeners produced by cyanobacterial blooms. Drowned-river mouth lakes are transitional zones between a lake and an inflowing river and have unique physical and hydrological dynamics^19^. West Michigan (USA) contains many drowned-river mouth lakes with histories of cyanobacteria blooms^20^,^21^. Studies of cyanotoxins in these lakes have been rare despite their high recreational use. Muskegon Lake and Bear Lake are both drowned river mouth systems, and are listed as a Great Lakes Area of Concern and require the restoration of Beneficial Use Impairments related to ‘Eutrophication or Undesirable Algae’ and ‘Restrictions on Drinking Water Consumption’ for delisting^21^. Detailed information concerning the nutrient chemistry, phytoplankton community dynamics, and cyanotoxins are necessary for delisting. Muskegon Lake, located on the eastern shore of Lake Michigan, has a long history of anthropogenic impairment^22^. Bear Lake is a hypereutrophic, shallow drowned river mouth system^23^ and the research into the cyanotoxins of cyanobacteria blooms in Muskegon Lake and Bear Lake began in the summer of 2006^24^. The relative composition of microcystin differed between Bear and Muskegon Lakes despite their hydrologic connectivity. MC-LR and MC-RR percentage were equally abundant in Bear Lake, whereas MC-LR composed 54–87% of the total MC in Muskegon Lake^24^ suggesting the MC toxicity in the hypereutrophic Bear Lake is lower than mesotrophic/eutrophic Muskegon Lake due to the predominance of the more toxic MC-LR. Xie *et al*.^24^ hypothesized the greater percentage contribution of MC-LR than MC-RR was due to Muskegon Lake having a significantly lower summer temperature and different nitrogen chemistry than Bear Lake. However, the effect of temperature and other environmental factors on microcystin analog ratios in both lakes has not been evaluated *in situ*.

The aim of this study was to test, in natural ecosystems, the hypothesis that different N and P forms, alone or in combination with other environmental variables, influence the cyanobacterial community structure, the MC concentration, as well as the MC congener composition. Various forms of dialysis culture have been successfully used for studying a variety of phytoplankton species under laboratory as well as field conditions to investigate of species interactions and production of diffusible and non-diffusible products^25^,^26^. Such studies can allow the effects of environmental variables on microcystin production to be evaluated in the natural lake environment. We incubated dialysis bags containing Muskegon and Bear Lake’s phytoplankton in both lakes simultaneously to determine if differences in biotic and abiotic factors would influence MC production and congener ratios.

## Results

### Cyanobacterial assemblages

**Figure 1:**
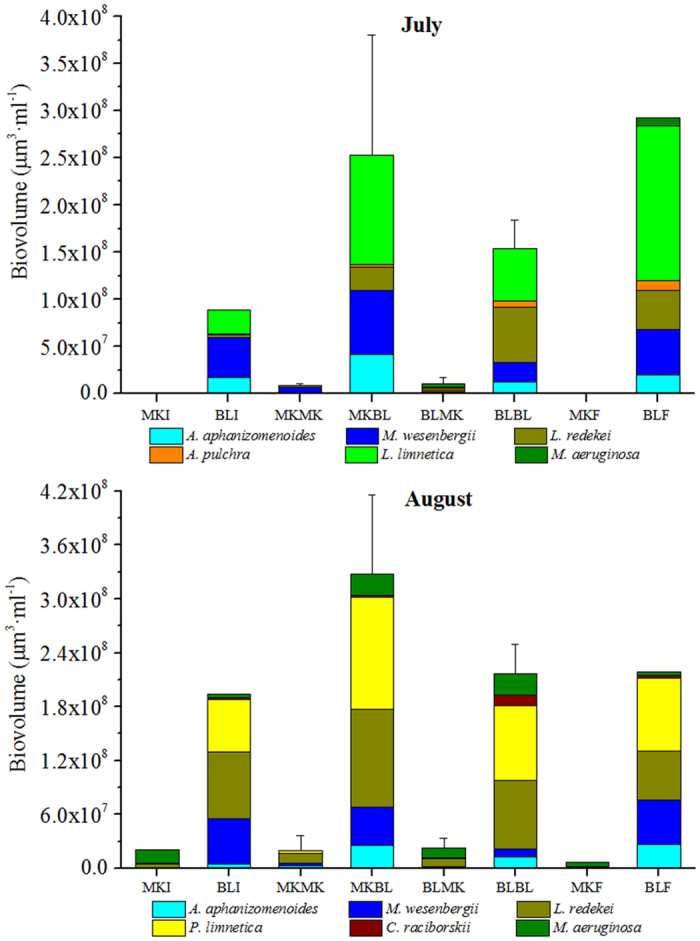
Cyanobacteria population composition in dialysis bag experiments (mean ± SD). (MKI: Muskegon Lake Initial; MKF: Muskegon Lake Final; MKMK: Muskegon Lake with Muskegon Lake water; MKBL: Muskegon Lake with Bear Lake water; BLI: Bear Lake Initial; BLF: Bear Lake Final; BLBL: Bear Lake with Bear Lake water; BLMK: Bear Lake with Muskegon Lake water. The taxonomic analyses were conducted with three replicates in July and five replicates in August.

Phytoplankton communities were dominated by cyanobacteria. In total, 23 cyanobacterial taxa were identified in the dialysis bags. The plankton was dominated by the same cyanobacterial species for all experiments. The greatest biovolume of cyanobacteria was noted in the dialysis bags incubated in Muskegon Lake with Bear Lake water (MKBL) (Fig. 1). In July, the six dominant cyanobacteria identified in all bags were *Aphanizomenon aphanizomenoides*, *Microcystis wesenbergii*, *Limnothrix redekei*, *Aphanocapsa pulchra*, *Lyngbya limnetica* and *Microcystis aeruginosa* (Fig. 1). The *A. aphanizomenoides* biovolume in the experiments with Bear Lake water (triplicates for MKBL1-3; BLBL1-3) was significantly higher than with Muskegon Lake water (MKMK1-3, BLMK1-3) (*p* < 0.005). However, there were no differences found between all dialysis bags with Bear Lake water (*p* = 0.160) for this taxon. The *M. wesenbergii* biovolume with Bear Lake water was significantly higher than with Muskegon Lake water (*p* < 0.010). No differences were found between the individual experiments with Bear Lake water for this taxon (*p* = 0.020). *L. redekei* was present at greater biovolumes in the experiments with Bear Lake water (MKBL: 3.8 ± 2.3 × 10^9^ μm^3^·mL^−1^; BLBL: 5.9 ± 2.9 × 10^9^ μm^3^·mL^−1^) when compared to the dialysis bags with Muskegon Lake water (BLMK: 2.1 ± 2.0 × 10^8^ μm^3^ mL^−1^; MKMK: 3.1 ± 0.9 × 10^8^ μm^3^·mL^−1^). *A. pulchra* (BLBL: 6.6 ± 0.5 × 10^8^ μm^3^·mL^−1^) and *L. limnetica* (MKBL: 1.7 ± 1.0 × 10^9^ μm^3^·mL^−1^) were major contributors during July but were not present in August. *M. aeruginosa* was only observed in the bags with Muskegon Lake water (MKMK: 1.2 × 10^7^ μm^3^·mL^−1^1.2; BLMK: 3.2 × 10^8^ μm^3^·mL^−1^) and in Bear Lake (9.1 × 10^8^ μm^3^·mL^−1^). No cyanobacteria cells were found in the initial lake water of Muskegon Lake (MKI) and only a minor population of *M. wesenbergii* (1.1 × 10^7^ μm^3^·mL^−1^) was found in the final lake water of Muskegon Lake (MKF).

In August (five replicates for the bags: MKBL1-5; BLBL1-5), the community structure shifted to *Planktolyngbya limnetica* and *L. redekei*, (Fig. 1) which became the dominant taxa (35.5% and 34.0%, respectively). Dominant species of the cyanobacterial communities throughout the two sampling periods in all the experiments were *P. limnetica* (maximum 1.7 × 10^12^ μm^3^·mL^−1^), *L. redekei* (maximum 1.5 × 10^12^ μm^3^·mL^−1^), *M. wesenbergii* (maximum 6.7 × 10^11^ μm^3^·mL^−1^), *A. aphanizomenoides* (maximum 4.9 × 10^11^ μm^3^·mL^−1^), and *M. aeruginosa* (maximum 3.6 × 10^11^ μm^3^·mL^−1^). Between July and August, significant differences were observed for mean biovolume of *M. aeruginosa* (*p* = 0.010) and *L. redekei* (*p* = 0.040). Mean biovolumes of *A. aphanizomenoides* (*p* = 0.140) and *M. wesenbergii* (*p* = 0.510) were not significantly different between dates. *C. raciborskii* trichomes were found in experiments with Bear Lake water and the ambient Bear Lake water, with the greatest biovolume of 1.6 × 10^11^ μm^3^·mL^−1^ observed.

### Microcystin dynamics

**Figure 2:**
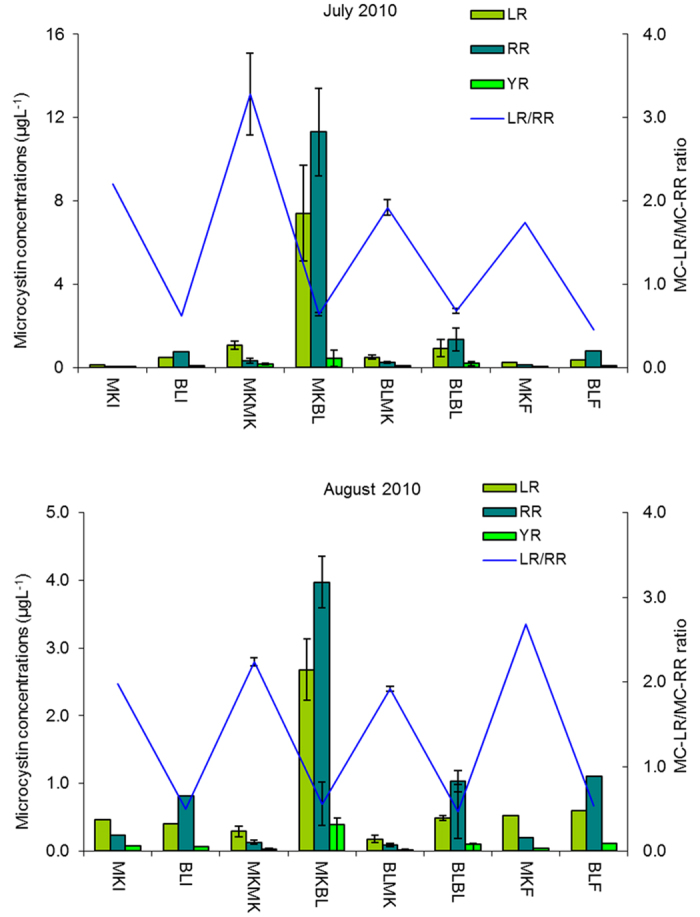
Microcystin analogue concentrations and MC-LR/MC-RR ratio in dialysis bags. (MKI; Muskegon Lake Initial, MKF; Muskegon Lake Final, MKMK; Muskegon Lake with Muskegon Lake water, MKBL; Muskegon Lake with Bear Lake water, BLI; Bear Lake Initial, BLF; Bear Lake Final, BLBL; Bear Lake with Bear Lake water, and BLMK; Bear Lake with Muskegon Lake water.)

In July, microcystins were detected in all samples analyzed in all dialysis bags but at lower concentrations in the experiments with Muskegon Lake phytoplankton (Fig. 2). The greatest total MC concentrations (20.1 ± 3.88 μg·L^−1^, range: 14.97–24.32 μg·L^−1^) were detected in bags incubated in Muskegon Lake with Bear Lake phytoplankton (MKBL1-3) (Fig. 2). MC-LR/MC-RR ratio of the bags initiated with Muskegon phytoplankton (MKMK; BLMK) and the ambient Muskegon Lake phytoplankton (MKI 7/16, MKF 7/22) were significantly higher than the other dialysis bags (BLBL; MKBL) and ambient Bear Lake water (BLI 7/16; BLF 7/22) (*p* = 0.010). Percent contributions of the MC-RR, MC-LR, and MC-YR congeners to total MC concentrations in the bags with Bear Lake phytoplankton (MKBL1-3, BLBL1-3) ranged from 53.8–60.6%, 32.4–38.4%, and 6.6–8.1%, respectively. Percent contributions of the MC-RR, MC-LR, and MC-YR congeners to total MC concentrations in the bags initiated with Muskegon Lake phytoplankton (MKMK1-3, BLMK1-3) ranged from 18.9–31.2%, 57.0–71.1%, and 8.2–11.9%, respectively. No statistically significant difference in MC-LR/MC-RR ratio was observed between the bags with Bear Lake phytoplankton and the ambient phytoplankton of Bear Lake (*p* = 0.100), but significant differences in the bags with Muskegon Lake phytoplankton and the ambient phytoplankton of Muskegon Lake (*p* = 0.020) were observed.

In August, the mean concentrations of total MC (7.04 ± 0.73 μg·L^−1^, range: 5.77–7.97 μg·L^−1^) in bags in Muskegon Lake initiated with Bear Lake phytoplankton (MKBL1-3) were also significantly higher than the MC in other bags (*p* < 0.001) (Fig. 2). MC-LR/MC-RR in the bags initiated with Muskegon phytoplankton (MKMK; BLMK) and the ambient phytoplankton from Muskegon Lake (MKI 8/16, MKF 8/22) were significantly higher than the other bags (BLBL; MKBL) and Bear Lake (BLI 8/16; BLF 8/22) (*p* < 0.001). Percent contributions of the MC-RR, MC-LR, and MC-YR congeners to the total MC concentrations in bags initiated with Bear Lake phytoplankton (MKBL1-3, BLBL1-3) ranged from 52.7–66.0%, 29.3–41.2%, and 3.82–8.61%, respectively. Percent contributions of the MC-RR, MC-LR, and MC-YR congeners to total MC concentrations in the bags initiated with Muskegon Lake phytoplankton (MKMK1-3, BLMK1-3) ranged from 28.2–36.0%, 54.4–66.2%, and 5.06–10.7%, respectively.

In both months, MC concentrations correlated with the biomass of *A. aphanizomenoides* (R^2^ = 0.312, *p* < 0.001, Spearman’s), *M. wesenbergii* (R^2^ = 0.121, *p* = 0.038), *L. limnetica* (R^2^ = 0.131, *p* = 0.030), but not correlated with *L. redekei* (R^2^ = 0.072, *p* = 0.115), *A. pulchra* (R^2^ = 0.070, *p* = 0.121), *P. limnetica* (R^2^ = 0.000, *p* = 0.984) and *M. aruginosa* (R^2^ = 0.004, *p* = 0.735), *C. raciborskii* (R^2^ = 0.041, *p* = 0.239) (Table 1). No MC-LA and CYN were detected throughout all the experiment.

### Environmental factors

Physicochemical parameters showed little temporal and spatial variation in Bear Lake and Muskegon Lake (Table 2). In both months, the SRP concentration was below the detection limit during the sampling period. The concentrations of nitrate (NO_3_-N) and ammonia (NH_3_-N) were higher in Muskegon Lake and the corresponding bags (MKMK; MKBL) than in Bear Lake and the corresponding bags (BLBL; BLMK) (*p* < 0.010 and *p* < 0.030, respectively). The MC concentrations were not correlated with the nitrate concentration (R^2^ = −0.422, *p* = 0.509) or ammonia concentration (R^2^ = −0.616, *p* = 0.150). The concentrations of TP and TN were higher in the Bear Lake and the bags initiated with Bear Lake phytoplankton (BLI; BLF; MKBL; BLBL) than Muskegon and the bags initiated with Muskegon Lake phytoplankton (MKI; MKF; MKMK; BLMK) (*p* < 0.010). The MC concentrations were correlated with the TN (R^2^ = 0.889, *p* < 0.001) and TP (R^2^ = 0.768, *p* = 0.020). There were no statistically significant differences noted between bags initiated with Muskegon Lake phytoplankton (MKMK; BLMK) and Bear Lake phytoplankton (BLBL; MKBL) during both months for Cl^−^ (*p* > 0.130), SO_4_^2−^ (*p* > 0.050), Hardness (*p* > 0.230), alkalinity (*p* > 0.310). MC concentrations were not correlated with Cl^−^ (R = −0.200, *p* = 0.880), SO_4_^2−^ (R = −0.224, *p* = 0.860), hardness (R^2^ = −0.200, *p* = 0.880) and alkalinity (R^2^ = 0.173, *p* = 0.910).[Table t3]

**Figure 3:**
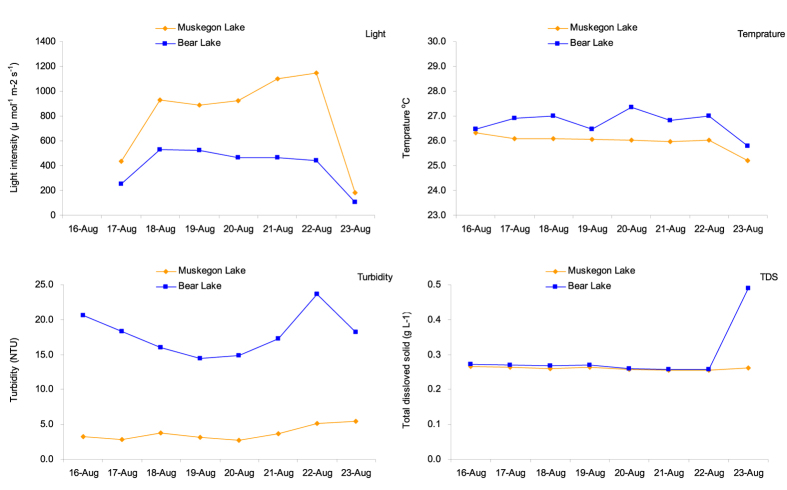
Environmental factor dynamics in Muskegon Lake and Bear Lake during the August dialysis bag experiments.

We measured the environmental factors in Bear Lake and Muskegon Lake in August (Fig. 3). Light intensity ranged from 181.5–1147.0 μmol·m^−2^·s^−1^ in Muskegon Lake and 107.5–526.8 μmol·m^−2^·s^−1^ in Bear Lake (Fig. 3). Statistically significant differences were noted between Bear Lake and Muskegon Lake for light intensity (*p* < 0.001), temperature (*p* < 0.001) and turbidity (*p* < 0.001), but not for TDS (*p* = 0.280) (Fig. 3).

## Discussion

Several research groups have studied how environmental parameters affect the dominance of cyanobacteria and total MC concentrations in lakes^11^,^13^,^27^. Also, some studies described the relationship of bloom community dynamics and the MC congener concentration and composition^6^,^13^,^28^. About MC congers, Tonk *et al*.^17^ suggested that the ratio of MC variants changed in response to differing light intensities; de Figueiredo *et al*.^29^ found out that higher temperatures enhanced MC-RR production, whereas lower temperatures favored MC-LR synthesis. While Monchamp *et al*.^13^ suggested that environmental factors did not appear to affect MC congener composition directly but there were significant associations between specific MC congeners and particular species. In our experiment, there was a significant difference in total MC concentrations between all the treatments. Total MC concentrations in the bags incubated in Muskegon Lake with Bear Lake water (MKBL) were significantly higher than the other treatments. No significant differences between the cyanobacteria biovolume in all the bags with Bear Lake water were observed and MC-LR/MC-RR ratios from the treatments with the corresponding lakes were similar during the study period.

According to previous studies^30^,^31^,^32^, MC production was correlated with algal species and cell growth. *M. aeruginosa* has been classified as a major MC producer in previous research^33^,^34^. In July, the greatest total MC concentrations (20.1 ± 3.88 μg·L^−1^, range: 14.97–24.32 μg·L^−1^) were found in bags without *M. aeruginosa* present. In addition, MC concentrations were not correlated with *M. aruginosa* biomass in both months, indicating that there were other cyanobacteria strains producing MC. MC concentrations were found to be correlated with *M. wesenbergii* in the current experiment. In term of MC production by *M. wesenbergii*, previous studies yielded contradictory conclusions. Henriksen^35^ found that *M. wesenbergii* was dominated in hepatotoxic *Microcystis* blooms of Danish lakes. While Watanabe^36^ concluded that *M. wesenbergii* has generally been considered as nontoxic. By both molecular and chemical methods, recent studies showed that *M. wesenbergii* lacked MC production genes in Germany and other European lakes^37^,^38^ and in China^39^. Also, in our early MC investigation in seven lakes of Michigan, the MC concentrations were not correlated with the biomass *M. wesenbergii* (unpublished data). Based on the literature findings in spite of the observed correlation, it was likely that *M. wesenbergii* was a nontoxic species in our experiments.

The traditional genus *Aphanizomenon* comprises a group of filamentous nitrogen-fixing cyanobacteria of which several members are able to develop blooms and to produce toxic metabolites (cyanotoxins), including hepatotoxins (microcystins), neurotoxins (anatoxins and saxitoxins) and cytotoxins (cylindrospermopsin)^40^. The species of *Sphaerospermopsis aphanizomenoides* isolated from Lake Oued Mellah was reported to contain MCs, namely four compounds displaying a retention time similar to that of MC-LA, LY, LW or LF in HPLC-PDA chromatograms^41^. In this study, MC concentrations correlated with the biomass of *A. aphanizomenoides* in both months indicating that *A. aphanizomenoides* is a potential MC producer. *A. aphanizomenoides* was considered to be salinity-tolerant^42^, requires high water temperature^43^, and the biomass of *A. aphanizomenoides* was found to be significantly related to the water temperatures^44^. This cyanobacterium has been detected in water bodies in several countries^44^ and has been expanding its range into more half regions of European^45^,^46^. *A. aphanizomenoides* has not been linked to MC production with the exception of a study also conducted in Bear Lake where the organism was listed as the dominant cyanobacteria species and a suspected MC producer^47^. In consideration of the strong statistical correlation between *A. aphanizomenoides* biovolume and MC production occurring in the same lake, our study assumes that *A. aphanizomenoides* may be a MC producer. Genetic studies still need to be performed to determine if toxin producing genes are present in this organism.

MC production also was influenced by environmental parameters^32^. Some studies suggested that the environmental parameters, i.e., phosphorus, nitrogen, temperature, light etc., affect the MC production and the growth of *M. aeruginosa* in continuous cultures, laboratory batch, or in the field^11^,^12^,^48^. Environmental parameters may affect MC concentrationsin two principal ways: regulating MC production by the toxigenic strains or regulating the population of MC-producing strains^49^. Sivonen^10^ indicated that MC production by *Oscillatoria agardhii* correlated with high nitrate concentration (0.42–0.84 mg·N/L) and low light intensity (12–95 μmol·m^−2^·s^−1^). While Jiang *et al*.^34^ suggested that light and iron had significant interactive effect on MC production. For *Microcystis* PCC 7806, Wiedner *et al*.^49^ indicated that the maximum MC concentrations were reached at light intensities of 40 μmol·m^−2^·s^−1^ but a decline in MC production and cellular MC content were observed by further increasing the irradiance during lab experiments. In addition, for *M. aeruginosa* W334, Hesse and Kohl^16^ found that celluar MC-LR concentrations decreased at a growth rate at 80 μmol·m^−2^·s^−1^, but for *M. aeruginosa* W368, MC-LR and MC-YR, cellular contents increased at 100 μmol·m^−2^·s^−1^. Yang *et al*.^50^ found out that MC production decreased significantly when the strain was exposed to UV-B radiation. For *P. agardhii*, Sivonen^10^ noted that higher MC concentrations were produced at lower irradiances (12 and 24 μmol·m^−2^·s^−1^) rather than at higher numbers (50 and 95 μmol·m^−2^·s^−1^). Monchamp *et al*.^13^ indicated that water temperature, TN, ammonium and DON can influence the cyanobacterial population structure, which resulted in the differences of the dominant MC congeners and the toxicity. It seemed that the diverse effects of light on the MC production depend on the cyanobacterial species and on the MC analogue. Currently, although opinions vary, MC production appears to be linked to N availability^27^,^51^,^52^ and functions to alleviate oxidative stress during high light conditions^53^,^54^.

In this study, MC-LR/MC-RR ratios varied with sample origin but not with lake of incubation, indicating that water temperature, light and turbidity were not the reasons for the difference of the MC-LR/MC-RR ratio. Van de Waal *et al*.^27^ studied how nitrogen pulse affect the MC variants of *P. agardhii* and found out MC-RR increased strongly, while MC-LR increased weakly after the nitrogen pulse. They speculated *Microcystis* and other MC-producing algae would respond similarly. In this study, we observed that the biovolume of *A. aphanizomenoides* followed the increase of MC production. *A. aphanizomenoides* is able to fix molecular nitrogen (diazotrophy) and in this study, we found low levels of NO_3_-N and NH_4_-N along with high levels of TN (Table 1). These numbers are typical for an environment in which N_2_ fixation takes place. Hence, it is possible that with fixed N_2_ made available for MC producing strains, both the overall MC content and the MC-LR/MC-RR ratio should be expected to change. With *A. aphanizomenoides* present, the limiting nutrient is supplied by N_2_ fixation may have resulted in the relative increase of MC-RR and MC-LR (Fig. 2). In Muskegon Lake water, the nutrient balance may not be suitable for N-fixation due to higher NO_3_-N concentrations since nitrate can suppress nitrogenase in some cyanobacterium^55^. Hence, the higher NO_3_-N concentrations were a possible factor for the increase of MC concentrations in the dialysis bags. Also, light was considered an important factor affecting MC production as light intensity can regulate the transcription of the MC-synthesizing gene^56^. In the present study, Muskegon Lake had lower temperature, higher light intensity, and lower turbidity than Bear Lake. Since the growth of *A. aphanizomenoides* requires higher water temperatures, the lower thermal profile observed in Muskegon Lake might not be conducive for the increase toxin production. In this study, the light intensity of Bear Lake (average: 397.2 μmol·m^−2^·s^−1^) was significantly lower than Muskegon Lake (800.1 μmol·m^−2^·s^−1^). Low-light conditions were generated by two main factors: water depth and turbidity^57^. Since we incubated all the dialysis bags in the same depth (1 m) of the two lakes, the higher turbidity of Bear Lake appears to be responsible for the lower light intensity. The high light intensity of Muskegon Lake appears be another reason for the increase of MC concentrations in the dialysis bags with Bear Lake water incubated in Muskegon Lake.

Oh *et al*.^58^ suggested that MC-LR/MC-RR ratio can increase with severe P-limited conditions. Sas *et al*.^59^ indicated that phytoplankton growth was P-limited if FRP was <10 μg·L^−1^ of the growing season. In this study, SRP of the two lakes and all the dialysis bags were less than 5 μg·L^−1^, TP in Muskegon Lake and the bags with Muskegon Lake water were all less than 50 μg·L^−1^, while TP in Bear Lake and bags with Bear Lake water were ~100 μg·L^−1^. The difference in bioavailable TP concentrations may be one reason for the dissimilarity of the MC-LR to MC-RR ratio of Muskegon Lake and Bear Lake. Furthermore, other factors which were not specifically investigated during the present study (e.g. turbulence, zooplankton predation) could also have an influence on the abundance of different microcystin congeners and we will do the further research in this field.

## Methods

### Experimental design

Experiments were conducted with water collected from Bear Lake and Muskegon Lake. Bear Lake has a surface area of 1.66 km^2^, an average depth of 2.14 m, and a maximum depth of 3.66 m^23^. Bear Lake discharges to Muskegon Lake through a narrow navigation channel at a rate of 0.9 m^3^/s and has a mean hydraulic residence time of 30 days^60^. Muskegon Lake is a mesotrophic/eutrophic, drowned river mouth system with a surface area of 16.6 km^2^ and an average depth of 7.1 m, with a maximum depth of 23 m^61^. Muskegon Lake discharges to Lake Michigan at a rate of 55.5 m^3^/s and has a mean hydraulic residence time of 25 days^61^. The Muskegon River accounts for 95% of the tributary inputs to Muskegon Lake^62^. Both lakes are well mixed^24^.

**Figure 4:**
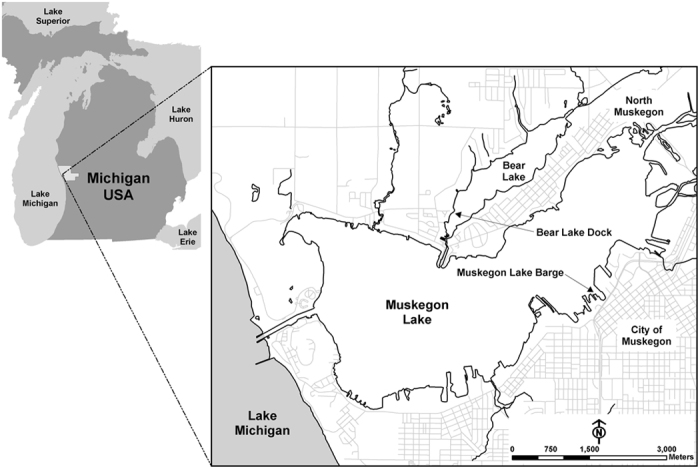
Sampling locations in Bear Lake Dock and Muskegon Lake Barge. This map was generated in ESRI ArcMap 10 (Environmental Systems Resource Institute, ArcMap 10 ESRI, Redlands, California, USA, http://www.esri.com/).

Dialysis bags were filled with lake water and phytoplankton from five meters away from Bear Lake Dock and 5 meters away from Muskegon Lake Barge (Fig. 4) at 1 meter depth in July 19^th^ 2010. All measurements occurred between 9:00 and 11:00 AM. The bags were constructed of Spectra/Por 5 dialysis tubing (12–14 K MWCO, 140 mm flat width; Spectrum Laboratories, CA) and contained approximately 500 ml of lake water and were completely sealed. Triplicate dialysis bags of water from each lake were attached to a support cage and incubated for 7 days in Bear Lake and Muskegon Lake at 1 m depth (the maximum depth of Bear Lake shore is 1.5 m). Dialysis bag samples were identified as MKMK (Muskegon Lake with Muskegon Lake water), MKBL (Muskegon Lake with Bear Lake water), BLBL (Bear Lake with Bear Lake water), and BLMK (Bear Lake with Muskegon Lake water). On August 16^th^, the samples were taken and incubated in the same location. To confirm the data of July was not random, we use 5 replicates of water from each lake at this time.

For chemical and biological analysis, water samples were collected near the support cages at the beginning (MKI and BLI, respectively) and end of the experiments (MKF and BLF, respectively). In addition, daily *in situ* measurements of Photosynthetically Active Radiation (PAR) were measured with a LiCor Li-193SA (spherical quantum sensor) and temperature, turbidity, and total dissolved solids (TDS) were measured with a YSI 6600. All *in situ* measurements were conducted adjacent to the dialysis bags at 1 m depth.

After the 7-day incubation period, the bags were mixed well prior to sampling and a 25 ml aliquot from each dialysis bag was withdrawn for phytoplankton analysis. The remaining water was stored immediately in a portable refrigerator (around 4 °C) and composited into a single sample for nutrient analysis.

### Chemical analysis

Three 100 ml aliquots from each dialysis bag were immediately placed on ice and returned to the lab for filtration on a 0.7 μm Whatman GF/F glass microfiber filter (Fisher Scientific cat # 09-874-64) and stored at −20 °C for cyanotoxin analysis. According to Fastner *et al*.^63^ and Dyble *et al*.^64^, toxin samples were lyophilized first and then sonicated in 75% aqueous methanol. MC analogues (MC-LR, MC-RR, MC-YR, MC-LA; Sigma-Aldrich) and cylindrospermopsin (CYN) (Sigma-Aldrich) analysis was performed by High-Performance Liquid Chromatography coupled Mass Spectrometry (HPLC/MS) using a Thermo Surveyor MSQ Single Quadrupole Mass Selective Detector and Thermo Spectrasystem gradient chromatographic system according to a method described by Barco *et al*.^65^. Total MC concentrations were reported as the sum of all congeners (HPLC/MS-Total).

Total Kjeldahl nitrogen (TKN-N) and ammonia (NH_3_-N) were analyzed on a BRAN+LUEBBE Autoanalyzer^66^. Nitrate (NO_3_-N), total phosphorus (TP-P), and soluble reactive phosphorus (SRP-P) were analyzed on an ion chromatograph (detections limit: 0.005 mg/L, Standard Methods 4100 C)^67^.

### Phytoplankton identification

Phytoplankton samples were preserved with 1% acidic Lugol’s solution. Algae were identified and enumerated utilizing a Nikon Eclipse TE200 inverted microscope^68^. At least 200–300 algal units (cells or filaments) were counted in all the samples. The cell volume of each species was calculated by applying the appropriate geometric formulae^69^. The detailing for cell density calculations please see Table 3.

### Statistical calculation

Statistical analyses were conducted with SPSS version 12.0.1 (SPSS, Inc. Chicago IL, USA). The non-parametric Wilcoxon sign test was used to evaluate MC concentrations differences between the bags and ambient samples in July and August as data were not normally distributed. Differences in cyanobacterial biovolume and MC concenration between the bags and ambient samples were examined with the non-parametric Wilcoxon sign test (a = 0.05). Statistical similarity was evaluated with the Mann Whitney U test (a = 0.05) and multiple correlations were performed with Spearman’s Rank-Order Correlation (a = 0.05). To test if the two months (July and August) had significantly different cyanobacterial assemblages, samples were analyzed with the nonparametric-analysis of similarity (ANOSIM, Clarke^70^). This method tests for significant differences (a = 0.05) between two or more groups using the rank order of the samples similarity matrix based on the Bray-Curtis similarity coefficient. To examine the differences between MC-LR/MC-RR ratio, the Mann Whitney U test was used (differences being significant at *p* < 0.05). To examine the differences between environmental factors, the Mann Whitney U test was used (differences being significant at *p* < 0.05).

## Conclusion

Our data suggest that differences in total phosphorus concentrations were a reason for the dissimilarity of the MC-LR/MC-RR ratio between Muskegon Lake and Bear Lake. The higher light intensity due to lower turbidity and NO_3_-N concentrations in Muskegon Lake were two factors contributing to an increase of total MC production.

## Additional Information

**How to cite this article**: Xie, L. *et al*. The impact of environmental parameters on microcystin production in dialysis bag experiments. *Sci. Rep.* **6**, 38722; doi: 10.1038/srep38722 (2016).

**Publisher's note:** Springer Nature remains neutral with regard to jurisdictional claims in published maps and institutional affiliations.

## Figures and Tables

**Table 1:** Spearman’s correlation results between MC concentrations and cyanobacterial species.

Parameters	MC concentrations	
	R^2^	Significance
*A. aphanizomenoides*	0.312**	*p* < 0.001
*M. wesenbergii*	0.121*	*p* = 0.038
*L. limnetica*	0.131*	*p* = 0.030
*A. pulchra*	0.070	*p* = 0.121
*L. redekei*	0.072	*p* = 0.115
*M. aruginosa*	0.004	*p* = 0.735
*P. limnetica*	0.000	*p* = 0.984
*C. raciborskii*	0.041	*p* = 0.239

^*/**^The correlation is significant at the 0.05/0.01 level (2-tailed).

**Table 2:** Chemical data (mean ± SD, n = 3 (July); n = 5 (August)) for dialysis bag experiments and ambient lake water (MKI: Muskegon Lake Initial; MKF: Muskegon Lake Final; MKMK: Muskegon Lake with Muskegon Lake water; MKBL: Muskegon Lake with Bear Lake water; BLI: Bear Lake Initial; BLF: Bear Lake Final; BLBL: Bear Lake with Bear Lake water; and BLMK: Bear Lake with Muskegon Lake water; MKI, MKF, BLI and BLF represented the ambient samples; “<”represented the concentration was below the limit of the detection).

Date	Stations	SRP mg·L^−1^	TP mg·L^−1^	NO_3_-N mg·L^−1^	NH_4_-N mg·L^−1^	TN mg·L^−1^	Cl^−^ mg·L^−1^	SO_4_^2−^ mg·L^−1^	Hardness mg·L^−1^	Alkalinity mg·L^−1^
July	MKI	<0.005	0.03	0.11	0.05	0.50	30	17	165	155
	BLI	<0.005	0.10	<0.01	0.02	1.15	58	14	135	114
	MKMK	<0.005	0.03 ± 0.001	0.23 ± 0.03	0.05 ± 0.003	0.70 ± 0.09	29 ± 1.0	19 ± 0.6	172 ± 10	153 ± 3
	MKBL	<0.005	0.09 ± 0.016	0.24 ± 0.04	0.03 ± 0.004	3.01 ± 0.97	29 ± 3.2	19 ± 0.6	167 ± 6	163 ± 6
	BLMK	<0.005	0.03 ± 0.003	0.05 ± 0.06	0.02 ± 0.005	0.70 ± 0.09	60 ± 3.5	14 ± 0.0	128 ± 3	105 ± 13
	BLBL	<0.005	0.08 ± 0.006	< 0.01	0.02 ± 0.002	2.23 ± 1.02	58 ± 4.7	15 ± 0.6	137 ± 6	113 ± 3
	MKF	<0.005	0.04	0.24	0.09	0.84	64	18	170	160
	BLF	<0.005	0.12	<0.01	0.02	1.34	34	14	129	110
August	MKI	<0.005	0.04	<0.01	0.03	0.63	29	19	170	152
	BLI	<0.005	0.09	<0.01	0.01	1.31	58	14	128	104
	MKMK	<0.005	0.05 ± 0.004	0.17 ± 0.03	0.05 ± 0.008	1.32 ± 0.10	31 ± 0.6	21 ± 0.6	171 ± 2	151 ± 2
	MKBL	<0.005	0.10 ± 0.003	0.16 ± 0.01	0.03 ± 0.008	2.86 ± 0.45	42 ± 5.8	20 ± 0.8	177 ± 8	166 ± 12
	BLMK	<0.005	0.04 ± 0.009	0.07 ± 0.01	0.02 ± 0.012	0.62 ± 0.13	62 ± 8.2	14 ± 0.5	129 ± 4	105 ± 4
	BLBL	<0.005	0.09 ± 0.020	< 0.01	0.01 ± 0.002	1.57 ± 0.10	59 ± 9.0	12 ± 1.5	129 ± 2	106 ± 8
	MKF	<0.005	0.06	0.17	0.11	0.64	28	21	172	152
	BLF	<0.005	0.09	<0.01	0.02	1.18	59	16	126	100

**Table 3 t3:** The detail calculations for biovolume of dominated cyanobacterial species in July for dialysis bag experiments and ambient lake water (MKI: Muskegon Lake Initial; MKF: Muskegon Lake Final; MKMK: Muskegon Lake with Muskegon Lake water; MKBL: Muskegon Lake with Bear Lake water; BLI: Bear Lake Initial; BLF: Bear Lake Final; BLBL: Bear Lake with Bear Lake water; and BLMK: Bear Lake with Muskegon Lake water; MKI, MKF, BLI and BLF represented the ambient samples; No any cyanobacterial cells was identified in MKI and MKF; Same method was used in August.

Site ID	Genera species	Cells	Volume Counted (mL)	Biovolume (1 × 10^3^ μm^3^·mL^−1^)
BLI	*A. aphanizomenoides*	A1	0.02168384	Cells/Volume
MKMK	*M. aeruginosa*	A2	0.04176147	Cells/Volume
MKBL	*L. redekei*	A3	0.01124347	Cells/Volume
BLMK	*A. pulchra*	A4	0.03854905	Cells/Volume
BLBL	*L. limnetica*	A5	0.02409315	Cells/Volume
BLF	*M. wesenbergii*	A6	0.01325124	Cells/Volume
